# *Pseudomonas mendocina* native valve infective endocarditis: a case report

**DOI:** 10.1186/s13256-016-1057-6

**Published:** 2016-10-04

**Authors:** Glenn J. Rapsinski, Jina Makadia, Nitin Bhanot, Zaw Min

**Affiliations:** 1 Temple University School of Medicine, Philadelphia, PA USA; 2 Division of Infectious Disease, Allegheny General Hospital, Allegheny Health Network, 420 East North Avenue, South Tower, East Wing, Suite 407, Pittsburgh, PA 15212 USA

**Keywords:** *Pseudomonas mendocina* infection, *Pseudomonas mendocina* septicemia, Endocarditis

## Abstract

**Background:**

Gram-negative microorganisms are uncommon pathogens responsible for infective endocarditis. *Pseudomonas mendocina*, a Gram-negative water-borne and soil-borne bacterium, was first reported to cause human infection in 1992. Since then, it has rarely been reported as a human pathogen in the literature. We describe the first case of native valve infective endocarditis due to *P. mendocina* in the USA.

**Case presentation:**

A 57-year-old white man presented with bilateral large leg ulcers, fever, and marked leukocytosis. His past medical history included gout and chronic alcohol use. *P. mendocina* was isolated from his blood cultures. A comprehensive review of *P. mendocina* infection in the literature was performed. A total of eight cases of *P. mendocina* infection were reported in the literature. More than two-thirds of the cases of *P. mendocina* septicemia were associated with native valve infective endocarditis. Thus, an echocardiogram was performed and demonstrated mitral valve endocarditis with mild mitral insufficiency. His leg wounds were debrided and were probably the source of *P. mendocina* bacteremia. Unlike *Pseudomonas aeruginosa*, *P. mendocina* is susceptible to third-generation cephalosporins. Our patient received a 6-week course of antimicrobial therapy with a favorable clinical outcome.

**Conclusions:**

Our reported case and literature review illuminates a rare bacterial cause of infective endocarditis secondary to *P. mendocina* pathogen. Native cardiac valves were affected in all reported cases of infective endocarditis, and a majority of affected heart valves were left-sided. The antibiotics active against *P. mendocina* are different from those that are active against *P. aeruginosa*, and they notably include third-generation cephalosporins. The outcome of all reported cases of *P. mendocina* was favorable and no mortality was described.

## Background

Infective endocarditis is a life-threatening infection of the endocardial surfaces of the cardiac valves. Gram-positive cocci, mainly *Staphylococcus aureus* and Streptococci, are the leading causes of infective endocarditis. Gram-negative rods and fungi are not common pathogens of infective endocarditis [1]. *Pseudomonas mendocina* causing infective endocarditis has rarely been reported in the literature. *P. mendocina* is a ubiquitous soil and water dwelling Gram-negative non-fermentative rod discovered in 1970 in Mendoza, Argentina [2]. For the first 22 years after its discovery, it was presumed to be non-pathogenic in humans. As a soil and water organism, the primary research into this organism looked at its abilities to remediate oils and organic chemicals. Little to no research was done into its pathogenicity or virulence factors. In 1992, the first case of infection in humans by *P. mendocina* was reported in Argentina in a 63-year-old man with infective endocarditis [3].

Here we present the first case of infective endocarditis from *P. mendocina* reported in the USA and perform a comprehensive review of the published literature of the infection.

## Case presentation

**Fig. 1:**
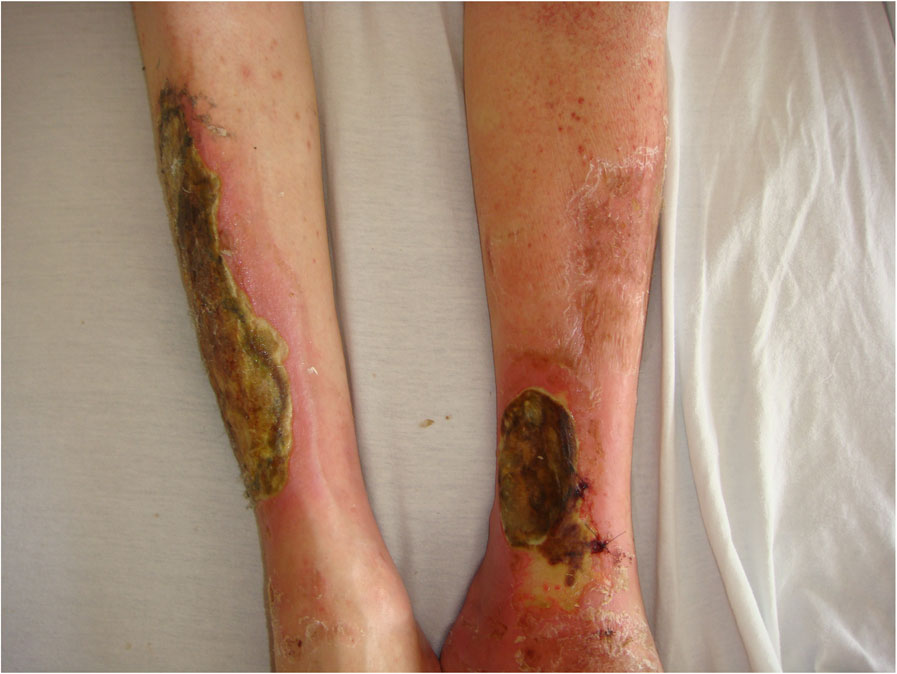
Two large ulcerated wounds on the patient’s legs

**Fig. 2:**
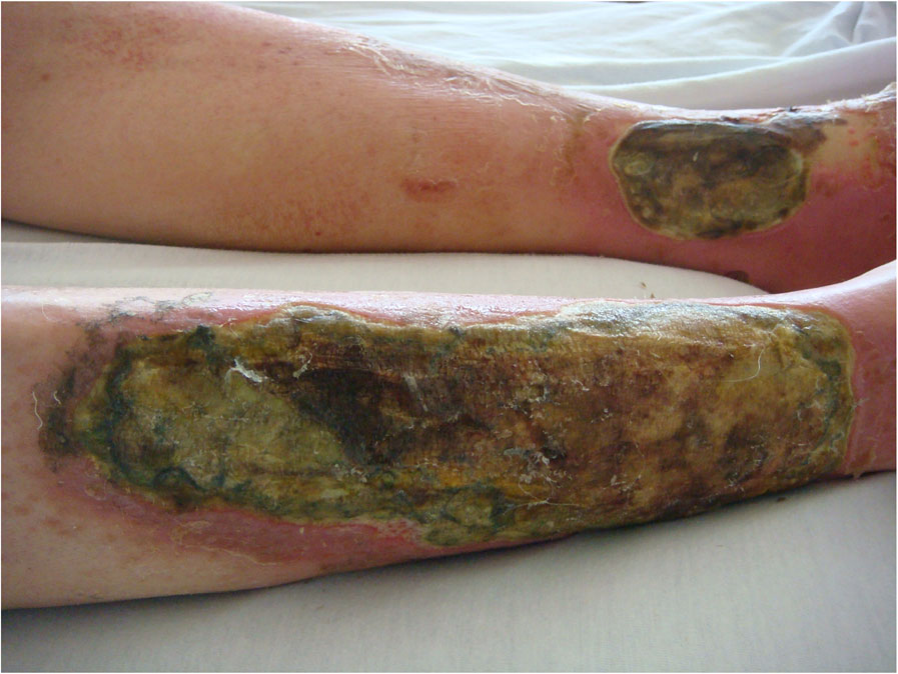
Two large ulcerated wounds on the patient’s legs

A 57-year-old white man presented to our institution with chief complaints of leg pain and a “rash” on his legs that he believed was a flare of his chronic and recurrent gout. He described severe leg pain, drainage of fluid from his leg wounds (Figs. 1 and 2), and difficulty walking. He could not recall trauma or injury to his legs. He denied any fever, but complained of some chills. The worsening of the drainage of his leg wound was what prompted him to seek help in an emergency room. His only known past medical history was gout. His social history was significant for at least six alcoholic drinks per day. He denied illicit drug use.

On physical examination, he was awake and oriented to time, place, and self. He was, however, in distress from his leg pain. His vital signs revealed a temperature of 36.6 °C (97.9 °F), heart rate of 90/minute, blood pressure of 172/96 mmHg, and respiratory rate of 16/minute with an oxygen saturation of 100 % on room air. The other remarkable physical findings included a gallop heart rhythm without heart murmurs, and jugular venous distention. There were two large cutaneous ulcerations over both his lower legs. On his right shin, the lesion measured approximately 25×8 cm. The wound on his left ankle was oval in shape and measured 8 cm in diameter (Figs. 1 and 2). Foul-smelling purulent drainage was noted on his right shin ulcer. Laboratory studies showed a marked leukocytosis of 24,000/mm^3^. X-rays of his tibia and fibula showed soft tissue edema, but no discreet osseous abnormalities. Blood cultures were drawn on admission due to leukocytosis and the extensive nature of the leg ulcerations. It was followed by empiric therapy with intravenous vancomycin and piperacillin-tazobactam.

During admission, he became febrile with a temperature of 38.5 °C (101.3 °F). A new systolic heart murmur (3/6 intensity) at the cardiac apex was noted on auscultation. Both sets of blood cultures grew *P. mendocina*. An antibiotic resistance profile was performed by a VITEK® analyzer (bioMérieux, France). The antibiotic susceptibilities of the *P. mendocina* isolate from our patient are summarized in Table 1. His antimicrobial therapy was modified to monotherapy with intravenous piperacillin-tazobactam in order to cover both *P. mendocina* and anaerobes in the leg ulcers due to putrid discharge and odor. On day 3 of hospitalization, a dermatology consult was requested for skin ulcer biopsy and culture to identify the source of *P. mendocina* septicemia. Two skin punch biopsies of his leg ulcers were obtained. His leg wounds were then debrided and dressed by our plastic surgery team.

**Table 1:** Antibiotic susceptibility profile of *Pseudomonas mendocina* isolated from our patient

Antibiotic	Minimal inhibitory concentration, susceptibility (μg/ml)
Ampicillin/Sulbactam	≥32, resistant
Cefazolin	32, resistant
Cefepime	≤1, susceptible
Ceftazidime	2, susceptible
Ceftriaxone	8, susceptible
Ciprofloxacin	≤0.25, susceptible
Gentamicin	≤1, susceptible
Piperacillin-tazobactam	≤4, susceptible

**Fig. 3:**
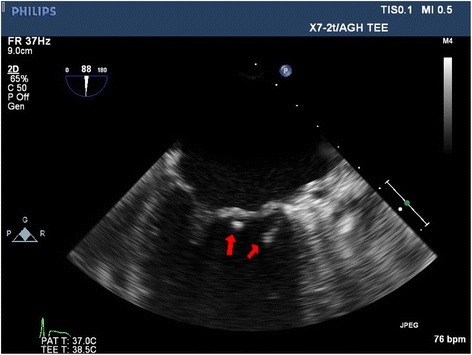
Transesophageal echocardiogram showed two vegetations on the patient’s mitral valve. *Red arrows* point to the vegetations

Cultures of these skin biopsies grew *Stenotrophomonas maltophilia*, but failed to isolate *P. mendocina* which may be secondary to the prior antibiotic therapy. *Stenotrophomonas maltophilia* was susceptible to trimethoprim-sulfamethoxazole and resistant to ceftazidime. Trimethoprim-sulfamethoxazole was added to his antibiotic regimen. Histopathology of his skin biopsy reported nonspecific acanthotic spongiotic parakeratosis with mixed lymphocytes and neutrophil infiltrates. The special stains of the skin tissues were negative for mycobacteria and fungi. The mycobacteria and fungal cultures of the biopsied cutaneous tissues remained negative for 8 weeks. A literature review showed there is an association of *P. mendocina* septicemia with infective endocarditis. Given a new cardiac murmur, a transesophageal echocardiogram was performed. The echocardiogram demonstrated two small mobile echo-densities (<1 cm) attached to his anterior mitral valve leaflet, suggestive of vegetations, with mild mitral regurgitation (Fig. 3). A diagnosis of *P. mendocina* native mitral valve infective endocarditis was made. Our cardiothoracic surgery team was consulted and recommended medical therapy given the small vegetations with trivial valvular insufficiency. Intravenous piperacillin-tazobactam therapy was continued, and he was discharged to a nursing facility to complete a 6-week course of antibiotic therapy. He returned for a 1-month follow-up and he had clinically improved and was able to ambulate with minimal assistance.

## Discussion

The genus *Pseudomonas* comprises at least 140 species [4]. Among them, *Pseudomonas aeruginosa* possesses an abundance of virulence factors and is regarded as the most important pathogen responsible for serious life-threatening infections in humans [4]. *P. mendocina*, a non-aeruginosa *Pseudomonas* species, rarely causes human infection.

*P. mendocina*, like other *Pseudomonas* species, is commonly found in soil, water, plants, and animals. A comprehensive review of the literature was performed by searching PubMed® and Google Scholar® using the terms “*Pseudomonas mendocina* infection” and “*Pseudomonas mendocina* endocarditis”. As shown in Table 2, the case reported here is the ninth reported case of human *P. mendocina* infection, but the first reported case in the USA [3, 5–11]. One case report was published in French and is included in this review [8]. All eight published cases are outside North America: three cases from Asia (Taiwan, Singapore), two cases from Europe (Denmark, France), two from the Middle East (Turkey, Israel), and one from Argentina. Of the eight reported cases, five were bacteremic [3, 5, 7–9]. The remaining three cases were non-bacteremic, and associated with bone and soft tissue infections [6, 10, 11]. Among the five patients with *P. mendocina* bacteremia, four cases (80 %) involved infective endocarditis [3, 5, 7, 8]. The affected cardiac valves were native in all four cases of infective endocarditis. The affected heart valves were predominantly left-sided. Valvular repair or replacement was required in two cases. The mechanism of predilection of valvular endocarditis by this pathogen is obscure. No mortality associated with the *P. mendocina* infection was reported.

**Table 2:** Published reports of *Pseudomonas mendocina* infection

Reference	Age (in years)/Gender	Country	Past medical history	Presenting complaint	Specimen site	Final diagnosis	Treatment	Valvular surgery	Outcome
Aragone *et al*., 1992 [3]	63/M	Argentina	Poliomyelitis, diabetes mellitus, aortic valve replacement, pacemaker	Fever and chills	Blood	Native mitral valve endocarditis	IV ceftriaxone + IV gentamicin (6 weeks), then po ciprofloxacin (2 weeks)	Not performed	Survived
Johansen *et al*., 2001 [5]	28/F	Denmark	Situs inversus, tetralogy of Fallot, ventricular septal defect closure, resection of pulmonary valve cusps	Abdominal pain, dyspnea, flu-like symptoms	Blood	Native tricuspid valve endocarditis	IV gentamicin + IV penicillin, then IV gentamicin + IV ampicillin, followed by po ofloxacin (7 weeks)	Tricuspid valve repair	Survived
Chi et al., 2005 [6]	65/M	Taiwan	Alcoholism, renal disease	Lower back pain	Deep spinal tissue	L4–L5 Spondylodiscitis	IV cefepime × 2 weeks, followed by po ciprofloxacin × 4 weeks	NA	Survived
Mert *et al*., 2007 [7]	36/M	Turkey	Mental retardation	Weight loss and fever	Blood	Native mitral valve endocarditis	IV ceftazidime + IV amikacin × 6 weeks	Mitral valve replacement	Survived
Suel *et al*., 2011 [8]	79/F	France	Atrial fibrillation, transient ischemic attack, hypertension	Fever	Blood	Native aortic valve endocarditis	IV piperacillin + IV gentamicin × 6 weeks	Not performed	Survived
Nseir *et al*., 2011 [9]	31/M	Israel	Nil	Fever and chills	Blood	Bacteremia (no vegetation on transthoracic echocardiogram)	IV gentamicin × 4 days + po ofloxacin × 2 weeks	NA	Survived
Howe *et al*., 2013 [10]	86/F	Singapore	Osteoporosis	Atraumatic left thigh pain	Periosteal Biopsy	Femur osteomyelitis	Not mentioned	NA	Survived
Chiu and Wang, 2013 [11]	34/M	Singapore	Nil	Motorcycle accident	Foot tissue	Foot wound infection (polymicrobial pathogens)	IV cefazolin + IV gentamicin, then po ciprofloxacin × 2 weeks, followed by po trimethoprim-sulfamethoxazole × 16 days	NA	Survived
Current Report	57/M	USA	Gout, alcoholism	Leg ulcers	Blood	Native mitral valve endocarditis	IV piperacillin-tazobactam × 6 weeks	Not performed	Survived

*F* female, *IV* intravenous, *M* male, *NA* not applicable, *po* per oral

The source of *P. mendocina* infection was not identified in most reported cases. Nseir and colleague’s speculation on the source of infection is fascinating [9]. They found that the patient had a peculiar habit of allowing a bird to drink water from his mouth. *P. mendocina* was isolated both from the tap water and the bird’s drinking water. In our case, our patient had already received intravenous antibiotics for 3 days when his leg wound cultures were obtained. His skin tissue cultures failed to grow *P. mendocina* because of the inhibitory effect of antibiotics on the bacterial growth. Thus, we postulated that the leg wound ulcers were the likely nidus of the infection.

Because *P. mendocina* is not a common human pathogen, little knowledge exists about the antimicrobial resistance patterns of this organism. This pathogen was reportedly susceptible to ampicillin in some reported cases [5, 7]. However, ampicillin or ampicillin-sulbactam is not tested in the antibiotic susceptibility of non-aeruginosa *Pseudomonas* species by the US Clinical and Laboratory Standards Institute (CLSI) [12]. The third-generation cephalosporins (cefotaxime, ceftriaxone, cefoperazone, ceftizoxime) are included in the susceptibility testing and recommended as a treatment of choice if susceptible [4, 12]. Other traditional “anti-*Pseudomonas aeruginosa*” antibiotics, including ceftazidime, cefepime, piperacillin-tazobactam, aminoglycosides, carbapenems, and ciprofloxacin, show an excellent activity against most strains of *P. mendocina* [4, 12]. In one case report, Mert *et al*. reported that their isolate of *P. mendocina* was susceptible to all tested antibiotics (ampicillin, ceftazidime, cefepime, piperacillin-tazobactam, imipenem, and gentamicin) [7]. Aragone *et al*. found ampicillin and cephalothin resistance in their isolate [3]. Resistance to ampicillin and ampicillin-sulbactam was demonstrated in the isolates from a foot wound by Chiu and Wang [11]. In a case of spondylodiscitis, the isolated *P. mendocina* pathogen from deep spinal tissue was only resistant to trimethoprim-sulfamethoxazole [6]. Nseir *et al*. identified ceftriaxone and aztreonam resistance in their isolate [9]. The other cases in Table 2 failed to report antibiotic resistances of their *P. mendocina* isolate. The present isolate in our patient was resistant to ampicillin-sulbactam and first-generation cephalosporins. While these isolates indicate a low level of antibiotic resistance in this organism, the minimal numbers of cases preclude an in-depth analysis of antibiotic resistance trends in this organism.

## Conclusions

In summary, there were only eight cases of *P. mendocina* human infections reported in the literature. Thus, there is a limitation to draw major conclusions from these cases. The actual incidence of *P. mendocina* infection is likely to be underestimated as well as underreported. All patients with *P. mendocina* infection survived. Most cases were noticeably associated with native valve infective endocarditis. The preponderance of *P. mendocina* for causing endocarditis warrants a discussion of echocardiography for patients with *P. mendocina* septicemia. Further research and clinical studies are required to identify the underlying mechanism and pathogenicity of this association. Although it is a relatively susceptible pathogen, we would not advocate using ampicillin, ampicillin-sulbactam, or the first- and second-generation cephalosporins as first-line therapy in a serious infection even if reported to be susceptible. The third-generation cephalosporins are acceptable and recommended in the treatment of *P. mendocina* infection. Guidance from antimicrobial susceptibility profiles in accordance with CLSI standards is always helpful for the optimal therapy with favorable clinical outcome.
